# Exploration of the Optimal Desmopressin Treatment in Children With Monosymptomatic Nocturnal Enuresis: Evidence From a Chinese Cohort

**DOI:** 10.3389/fped.2020.626083

**Published:** 2021-01-25

**Authors:** Jiaojiao Liu, Jiajia Ni, Qianfan Miao, Chunyan Wang, Fang Lin, Qi Cao, Wei Guo, Xue Yang, Xiaolu Ji, Yihui Zhai, Yunli Bi, Qian Shen, Hong Xu

**Affiliations:** ^1^Department of Nephrology, Children's Hospital of Fudan University, National Pediatric Medical Center of China, Shanghai, China; ^2^Department of Urology, Children's Hospital of Fudan University, Shanghai, China

**Keywords:** monosymptomatic nocturnal enuresis, children, desmopressin, efficacy, treatment

## Abstract

**Objectives:** Nocturnal enuresis (NE) is a common pediatric condition, and desmopressin (dDAVP) is a first-line therapy for NE. The standard initial dosage of dDAVP is 0. 2 mg/day, and most guidelines recommend that the dose should be increased at 0.2 mg increments until dryness is achieved or to the maximal recommended dose. However, previous evidence has shown that this strategy seems insufficient to further improve efficacy and results in unnecessarily high doses for some patients. Our study aimed to assess the efficacy of our modified dDAVP treatment regimen in children with MNE in China and evaluate predictive factors associated with the dDAVP response.

**Methods:** All MNE patients at the Department of Nephrology at Children's Hospital of Fudan University from January to December 2019 were prospectively and consecutively enrolled. dDAVP treatment comprised a dose titration period and a 3-month maintenance period. The efficacy of dDAVP was assessed according to the latest International Children's Continence Society criteria at the end of the study. Predictive factors were evaluated by logistic regression analysis.

**Results:** Overall, 322 MNE patients were enrolled in our study, and 225 (69.9%) completed the study. The intention to treat analysis showed that the overall dDAVP response rate was 69.9%: among these patients 32.3% were complete responders, and 37.6% were partial responders. At the end of the study, 194/225 (86.2%) patients received a final dose of 0.2 mg, 24/225 (10.7%) patients received a final dose of 0.3 mg, and 7/225 (3.1%) patients received a final dose of 0.4 mg. Multivariate analysis showed that patients requiring lower doses to achieve responses were significantly more likely to experience complete response during the maintenance period [odds ratio (OR)=9.683; 95% confidence interval (CI), 2.770–33.846].

**Conclusions:** Our results indicate that the dDAVP treatment regimen provides a comparable efficacy to the international conventional treatment regimen with a lower overall dose. Low-dose responders were likely to achieve a complete response without increasing the dose; in these cases, the maximum dose might not be necessary.

## Introduction

Nocturnal enuresis (NE) is defined as the involuntary voiding of urine at night in children aged 5 years or older (1). NE is a common disorder in children, with a prevalence of 15–20% in 5-year-olds, 7% in 7-year-olds, 5% in 10-year-olds, 1–2% in 15-year-olds and ~1% in adults (2, 3). Patients without daytime symptoms are categorized as having monosymptomatic nocturnal enuresis (MNE) and desmopressin (dDAVP) is a first-line therapy for patients with MNE. The standard recommended dose for treating MNE is 0.2–0.4 mg tablets, and the literature strongly supports that the dose should be gradually increased until dryness is achieved (4–6). For partial responders and non-responders, dDAVP is often increased by 0.2 mg increments to the maximal recommended dose until dryness is achieved. However, previous evidence has shown that this strategy seems insufficient to further improve the efficacy and even results in unnecessarily high doses for some individuals (7, 8). Considering the characteristics of the Chinese population and the availability of 0.1 mg dDAVP tablets in the Chinese marketplace, a treatment regimen that starts at 0.2 mg and increases doses by 0.1 mg every 2 weeks for non-responders until a complete response is achieved is suggested in China, although the efficacy of this strategy has not been well-evaluated. In this study, we aimed to explore the dosage plan of desmopressin in children with MNE in China and evaluate predictive factors associated with the dDAVP response.

## Patients and Methods

All the patients diagnosed with MNE at the Department of Nephrology of Children's Hospital of Fudan University from January 2019 to December 2019 were prospectively and consecutively enrolled. The inclusion criteria were age ≥5 years and diagnosed with MNE. To select children with MNE, patients were excluded if they had any daytime lower urinary tract symptoms (urgency, frequency and daytime wetting), recurrent urinary tract infections, untreated constipation, or neurogenic bladder. Additionally, disagreement with our treatment protocol and a history of any treatment for MNE within the preceding 3 months were also excluded. The pre-study evaluation included the medical history, physical examination, and laboratory tests such as urine analysis. Furthermore, a 4-day diurnal frequency-volume chart and 7-day nocturnal records were completed before treatment. The expected bladder capacity (EBC) for the child's age was calculated using the following formula: (age in years) ^*^ 30) + 30 mL (9). Nocturnal polyuria is defined as nocturnal urine production on a night with enuresis of at least 130% of the EBC for the child's age. Low bladder capacity (LBC) is defined as the highest micturition volume that is <65% of the EBC for the child's age. The study was approved by the ethical committee of Children's Hospital of Fudan University. Written informed consent was obtained from their patients or legal guardians.

### Study Design

The treatment comprised a dose titration period and a 3-month maintenance period. The 0.1 mg tablet formulation of desmopressin was used in this study. Titration approach: All the enrolled patients started with 2 × 0.1 mg desmopressin tablets for 2 weeks. If the response was not a ≥50% reduction over baseline, the dose was increased by 0.1 mg at 2-week intervals until a response or until reaching the maximum dose of 0.4 mg. For responders, treatment continued at the same dose for a 3-month maintenance period, and non-responders stopped treatment. Complete responders after 3 months of treatment underwent an abrupt withdrawal and were followed up for 6 months. All the enrolled children were provided clear instructions: They had to take dDAVP at least 2 h after the evening meal and 1 h before bedtime, restrict fluid intake 1 h before and 8 h after taking the medicine, and empty the bladder before going to sleep. Clinical follow-up was performed at 2-week intervals during the titration period, at 1-month intervals during the maintenance period to assess the treatment response, compliance, and severe adverse events. Patients were followed for 6 months after treatment withdrawal. The efficacy of dDAVP was assessed at the end of the maintenance period. Predictive factors including age, sex, body weight, family history, bladder capacity, nocturnal polyuria, number of wet nights, and treatment dosage, were evaluated by logistic regression analysis.

### Definitions of Treatment Outcomes

During the dose titration period, patients who achieved a ≥50% reduction over baseline were considered responders. Low-dose responders were defined as patients who achieved a response with 0.2 mg of dDAVP, and high-dose responders were defined as patients who achieved a response with 0.3 or 0.4 mg of dDAVP. Treatment outcomes were assessed using the latest ICCS criteria: no response (NR) was defined as less than a 50% decrease in the number of wet nights per week compared with baseline, partial response (PR) was defined as a 50–99% decrease, and complete response (CR) was defined as a 100% decrease. Relapse was defined as more than one symptom recurrence per month (9).

### Statistical Analysis

Statistical analyses were performed using SPSS 25.0. Descriptive statistics were expressed as the means±standard deviation (SD), with percentages for categorical variables. *T* tests, chi-square tests, and Fisher's test were used to compare the differences between variables. Logistic regression analysis was used to identify predictive factors of CR. *P* < 0.05 was considered statistically significant.

## Results

### Study Overview

**Figure 1:**
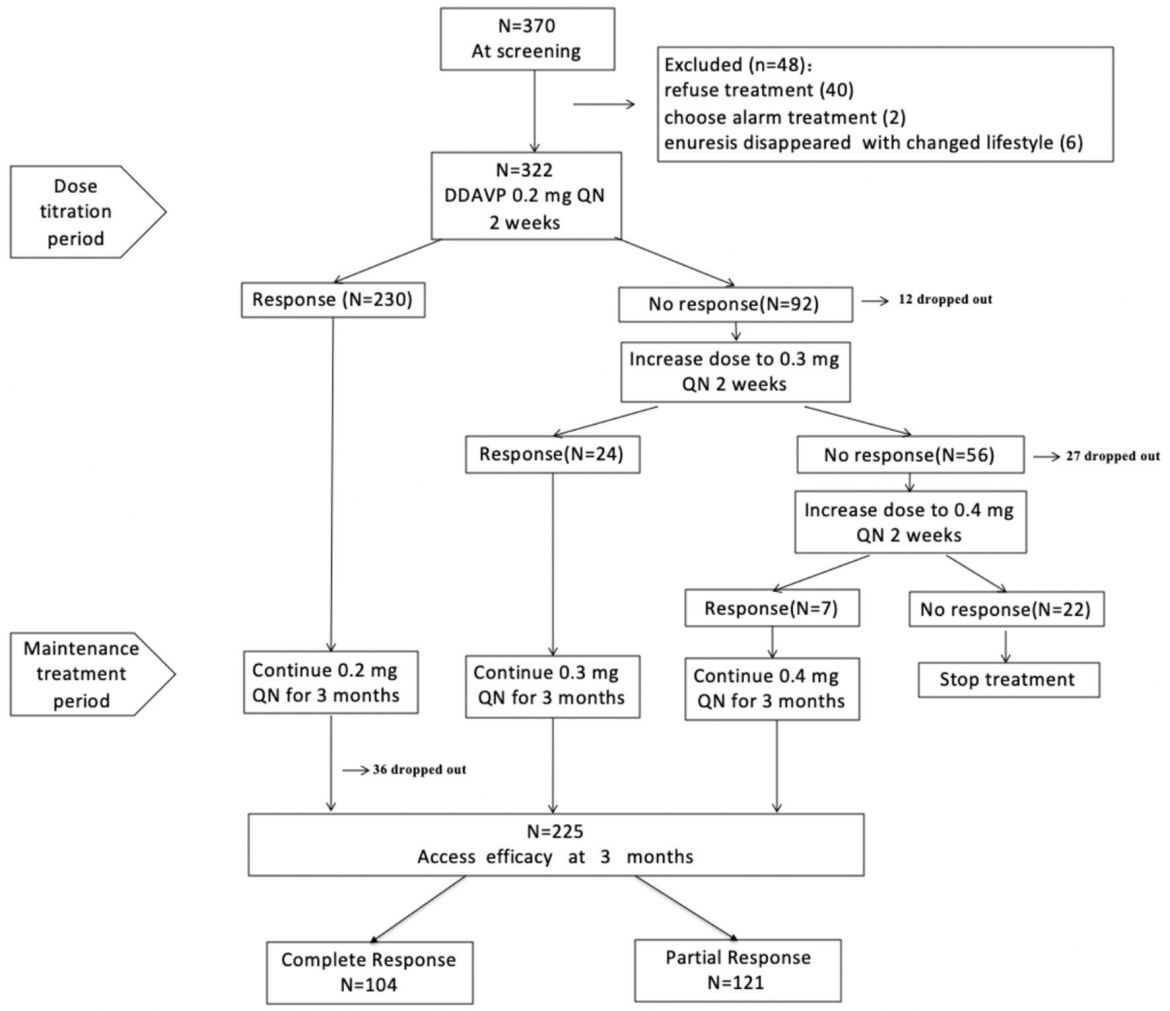
Study flow chart.

Overall, 370 MNE patients were screened from 15 provinces and municipalities in China (57% local patients and 43% outlying medical patients), and 322 children were enrolled in our study. In total, 283 patients (87.9%) completed the titration period, and 225 (69.9%) finished the study (Figure 1). The reasons for premature withdrawal from the study were as follows: lost to follow-up (34 patients, 10.6%), declined to continue treatment (30 patients, 9.3%), lack of efficacy (22 patients, 6.8%), and violated protocol (11 patients, 3.4%). Table 1 shows the baseline demographics and disease characteristics.

**Table 1:** Baseline demographic and disease characteristics.

**Sex**, ***n*** **(%)**	
Male Female	191 (59.3%) 131 (40.7%)
**Age (years)**	
Mean ± SD Range	8.29 ± 2.37 5–17
**Body weight (kilograms)**	
Mean ± SD Range	28.30 ± 9.63 14–63
**Family history**, ***n*** **(%)**	
Yes No NA	98 (30.4%) 184 (57.1%) 40 (12.4%)
**Nocturnal polyuria**, ***n*** **(%)**	
Yes No NA	206 (64.0%) 110 (34.2%) 6 (1.8%)
**LBC**, ***n*** **(%)**	
Yes No NA	109 (33.9%) 207 (64.3%) 6 (1.8%)
**Wet nights/week**	
Mean ± SD Range	5.92 ± 1.63 2–7

*LBC, Low bladder capacity*.

### Patient Disposition and Treatment Outcomes

#### Dose Titration Period

Initially, 230/322 patients (71.4%) responded to 0.2 mg dDAVP treatment, whereas 92 patients (28.6%) failed to respond and were recommended an increased dose according to the protocol. Overall, 80/92 patients (70%) agreed to receive a higher dose. Increasing the dose from 0.2 to 0.3 mg resulted in a response in 24/80 patients (30%) who did not respond to 0.2 mg, whereas the remaining 56 patients remained non-responsive. Then, 29/56 patients increased the dose from 0.3 to 0.4 mg, and 7/29 patients (24%) achieved a response. Hence, 261 responders entered the maintenance therapy period.

#### Maintenance Treatment Period

During the maintenance treatment period, 36 patients were lost to follow-up, and 225 patients completed the study. Intention-to-treat analysis was used to assess the effect of dDAVP, and the results showed that the overall response rate was 69.9%; among these patients, 32.3% were CRs, and 37.6% were PRs. At the end of the study, 194/225 (86.2%) patients received a final dose of 0.2 mg, 24/225 (10.7%) patients received a final dose of 0.3 mg, and 7/225 (3.1%) patients received a final dose of 0.4 mg.

#### Subgroup Analyses

Responders were divided into two groups based on the treatment dosage. The CR rate was significantly higher in the low-dose group (0.2 mg) than in the high-dose (0.3 mg or 0.4 mg) group (52.1 vs. 9.7%; *p* < 0.001) (Table 2). In a subgroup with 100% polyuric patients, the CR rate was significantly higher in the low-dose group (0.2 mg) than in the high-dose (0.3 mg or 0.4 mg) group (60.2 vs. 6.25%; *p* < 0.001) (Supplementary Table 1).

**Table 2:** Subgroup analysis of treatment outcomes.

**Groups**	**CR**	**PR**	**Total**	**CR rate**	P^*^
Low-dose responders	101	93	194	52.1%	<0.001
High-dose responders	3	28	31	9.7%	
Total	104	121	225	46.2%	

*CR, complete response; PR, partial response*.

* *Compared by Fisher's exact test, p < 0.001*.

#### Predictors of CR

Multivariate analysis indicated that patients who achieved a response with a lower dose were significantly more likely to experience a CR than those who required a higher dose of dDAVP to achieve a response [odds ratio (OR) = 9.683; 95% confidence interval (CI), 2.770–33.846] (Table 3).

**Table 3:** Logic regression analyses of factors predicting CR.

	**Multivariate analysis**		
**Variables**	**OR**	**95% CI**	***P***
Age (1-year increase)	0.970	0.806–1.169	0.751
Male	1.059	0.574–1.956	0.854
Body weight	0.993	0.949–1.038	0.742
Family history	0.951	0.493–1.835	0.880
Wet nights/week (≤ 5)	0.822	0.430–1.574	0.555
LBC	0.773	0.383–1.560	0.472
Nocturnal polyuria	0.600	0.298–1.208	0.152
Achieve response with lower dose	9.670	2.766–33.808	<0.001*

*CR, complete response; LBC, low bladder capacity*.

#### Relapse

After drug discontinuation, 45/104 (43.3%) complete responders experienced relapse during 6 months follow up.

### Assessment of Safety

No severe side effects related to dDAVP treatment were recorded.

## Discussion

dDAVP is a first-line treatment for NE. The efficacy and safety of dDAVP treatment in enuresis patients are well-documented (8, 10, 11). Previous trials have shown that ~60%–70% of patients with MNE respond to dDAVP therapy (7, 10–12). Approximately 30% achieved a CR, and 40% achieved a PR (6). Our study observed a CR in 32.3% and a PR in 37.6% of patients with a lower dDAVP dose (86% treated with 0.2 mg), similar to the reported treatment outcome (most were treated with the maximum dose). One previous study (13) showed that only 16% required 0.4 mg, and 9% of patients required 0.6 mg to achieve a 50% reduction from baseline. However, 87% of these patients received the maximum dose, indicating an unnecessary increase in the dDAVP dose for these patients, with similar efficacy between the lower dose and higher dose. Our data with a lower dosage showed similar response rates comparable to those previously reported. Notably, our titration approach used to obtain a response (only non-responders received an increased dose) was different from the conventional approach. The literature has recommended that the standard initial dosage of dDAVP tablets is 0.2 mg daily for treating NE, and the widely used titration approach increases the dose by 0.2 mg (1 tablet) every 2 weeks until a CR is achieved to maximize the chances of treatment success. This finding illustrates the superiority of our modified titration approach for optimizing the dose of dDAVP with an acceptable response.

Given the small body size of Chinese children and availability of 0.1 mg tablets in the marketplace, we used a modified titration approach (increasing the dose by 0.1 mg) to optimize the treatment dosage and explore whether a lower dose of dDAVP is effective for the Chinese population. In our study, increasing the dose from 0.2 to 0.3 mg during the titration period resulted in a response in 30% of 0.2 mg non-responders, and increasing the dose from 0.3 to 0.4 mg resulted in a response in 24.1% of 0.3 mg non-responders. The results of our study showed the superiority of the modified titration approach in optimizing the dosage for individuals and avoiding an unnecessarily high dose for some patients. Hyponatremia is a rare but severe side effect. Increasing the dose of dDAVP will most likely prolong the duration of action and rarely leads to a better response but entails a risk of hyponatraemia. Higher doses should be carefully and rationally used if persistent diluting capacity is documented in the morning in a specialized enuresis center to minimize the risk of hyponatraemia (14).

Kruse et al. (15) reported that most of full responders (59%) needed the lowest dose of dDAVP, which indicated that full responders seldom required the maximum dose of dDAVP to become dry. Similar to these findings, our results showed that low-dose responders had a higher rate of CR than high-dose responders (52.1 vs. 9.7%). These findings indicate that children who initially respond to dDAVP are more likely to achieve a CR during maintenance treatment than high-dose responders. Thus, we conclude that in some cases, the maximum dose of medication is not necessary and that some patients may achieve an acceptable response equivalent to that observed with higher doses.

Previous studies have shown that several factors, such as larger bladder capacities, nocturnal polyuria, older age and fewer wet nights at baseline, are associated with a better response (15–17). In our study, logistic regression analysis showed that age, sex, body weight, family history, bladder capacity, nocturnal polyuria, and number of wet nights were not predictive factors of the response to dDAVP. Only the initial response to low-dose dDAVP was a positive predictor of greater therapeutic success. Subgroup analysis indicated that low-dose responders were more likely to achieve a CR than high-dose responders. This finding suggests that in practice, clinicians can predict the treatment efficacy based on the initial response, increasing patients' confidence in the success of treatment.

Regarding the degree of response, our results showed that 32.3% of patients achieved a CR, and 37.6% of them achieved a PR. According to the literature (18, 19), approximately one-third of MNE patients had detrusor overactivity during sleep, likely explaining their partial or non-response to dDAVP despite receiving an adequate dosage. In our study, 33.9% of children with MNE had a low bladder capacity. This finding suggests that children in our study may not have been exclusively monosymptomatic, and the selection bias of including patients with undetected daytime symptoms might have partially limited the response to dDAVP. Furthermore, many other possible reasons could explain the suboptimal treatment response. On the one hand, besides the blunted circadian rhythm of vasopressin secretion, the altered circadian cycle of the antidiuretic hormone or influence of vasoactive hormones and prostaglandins might play a role in nocturnal polyuria, particularly in desmopressin-resistant patients (20). On the other hand, the poor bioavailability with large intra-and interindividual variances in plasma concentration should be considered (21).

Several other strategies to optimize the response to dDAVP, such as selecting the most appropriate formulation (most often the oral lyophilizate formulation), ensuring the optimal timing of administration and considering the possible impact of meals, ensuring fluid restriction before and after dDAVP administration, considering the impact of body weight, ensuring patients are adherent to treatment and administration recommendations, and considering a structured withdrawal strategy (22). For patients with confirmed MNE who have been identified as likely to benefit from dDAVP treatment, considering those important factors may be appropriate to further improve the efficacy.

Some limitations of our study must be acknowledged. First, this was a prospective cohort study, with no matched conventional treatment protocol group available to compare the efficacy. Furthermore, some patients who did not comply with the protocol during the titration period were not included in this study, possibly leading to an underestimating of the proportion of patients who require high-dose treatment. The Children's Hospital of Fudan University is one of the National Pediatric Medical Centers in China and focuses exclusively on providing pediatric care not only for local patients but also for medical patients from other regions. Although our patients came from multiple regions of China, this single center study might not represent the entire Chinese pediatric population. To further support our findings, more multicentre, high-quality randomized controlled trials that include head-to-head comparisons of different treatment regimens of dDAVP are recommended.

## Conclusion

Our results indicate that the dDAVP treatment regimen provides a comparable efficacy to the international conventional treatment regimen with a lower overall dose. Furthermore, our findings supported that low-dose responders are more likely to achieve a CR without increasing the dose; in these cases, the maximum dose of medication would be necessary.

## Data Availability Statement

The original contributions presented in the study are included in the article/Supplementary Material, further inquiries can be directed to the corresponding author/s.

## Ethics Statement

The studies involving human participants were reviewed and approved by Ethical committee of Children's Hospital of Fudan University. Written informed consent to participate in this study was provided by the participants' legal guardian/next of kin.

## Author Contributions

JL, JN, and QM contributed to the data analysis and drafted the manuscript. HX and QS contributed to the study design and critically revised the manuscript for important intellectual content. CW, FL, QC, WG, XY, XJ, and YB contributed to the patient follow-up and data collection. All authors have approved the final version of the manuscript to be published. Each author participated sufficiently in the work to be responsible for the content.

## Conflict of Interest

The authors declare that the research was conducted in the absence of any commercial or financial relationships that could be construed as a potential conflict of interest.
