# Supplementary material for: Exploration of the Optimal Desmopressin Treatment in Children With Monosymptomatic Nocturnal Enuresis: Evidence From a Chinese Cohort
Source: Front Pediatr. 2021 Jan 25;8:626083. doi: 10.3389/fped.2020.626083 (PMC7868531; doi:10.3389/fped.2020.626083)
Supplement: Supplementary file 1 [file Table_1.docx]

Supplemental Table 1. Subgroup analysis of treatment outcomes in polyuric patients

| Groups | | CR | PR | Total | CR rate | P value* |
| --- | --- | --- | --- | --- | --- | --- |
| Low-dose responders | | 74 | 49 | 123 | 60.2% | <0.001 |
| High-dose responders | | 1 | 15 | 16 | 6.25% |  |
| Total | 75 | | 64 | 139 | 53.9% |  |

CR, complete response; PR, partial response.

*Compared by Fisher’s exact test, p<0.001
